# BaroTac: Barometric Three-Axis Tactile Sensor with Slip Detection Capability

**DOI:** 10.3390/s23010428

**Published:** 2022-12-30

**Authors:** Gyuwon Kim, Donghyun Hwang

**Affiliations:** 1 Korea Institute of Science and Technology, Seoul 02792, Republic of Korea; 2 School of Mechanical Engineering, Korea University, Seoul 02841, Republic of Korea

**Keywords:** barometric tactile sensor, three-axis force tactile sensor, slip detection

## Abstract

Tactile sensors for robotic applications enhance the performance of robotic end-effectors as they ca n provide tactile information to operate various tasks. In particular, tactile sensors can measure multi-axial force and detect slip can aid the end-effectors in grasping diverse objects in an unstructured environment. We propose BaroTac, which measures three-axial forces and detects slip with a barometric pressure sensor chip (BPSC) for robotic applications. A BPSC is an off-the-shelf commercial sensor that is inexpensive, easy to customize, robust, and simple to use. While a single BPSC-based tactile sensor can measure pressure, an array of BPSC-based tactile sensors can measure multi-axial force through the reactivity of each sensor and detect slip by observing high frequency due to slip vibration. We first experiment with defining the fundamental characteristics of a single-cell BPSC-based sensor to set the design parameters of our proposed sensor. Thereafter, we suggest the sensing method of BaroTac: calibration matrix for three-axis force measurement and discrete wavelet transform (DWT) for slip detection. Subsequently, we validate the three-axis force measuring ability and slip detectability of the fabricated multi-cell BPSC-based tactile sensor. The sensor measures three-axis force with low error (0.14, 0.18, and 0.3% in the X-, Y- and Z-axis, respectively) and discriminates slip in the high-frequency range (75–150 Hz). We finally show the practical applicability of BaroTac by installing them on the commercial robotic gripper and controlling the gripper to grasp common objects based on our sensor feedback.

## 1. Introduction

Robotic end-effectors were made to handle tasks in diverse environments; therefore, numerous efforts to enhance their grasping accuracy and stability have attracted the attention of many researchers [1,2,3]. Specifically, end-effectors often miss the grasped object when interacting with various objects with varying properties. For instance, when handling fragile or vulnerable objects with a minimum force so as not to damage them, they are very likely to miss the grasped object, leading to the failure of the task. Adopting a tactile sensor is one attempt to handle this issue due to its functionalities in localizing the contact force [4,5,6], measuring multi-axial forces [7,8,9], and detecting dynamic events between end-effectors and grasped objects [10,11,12]. Among the various functionalities, tactile sensors capable of measuring forces in multi-axis and detecting slip can highly improve performance accuracy and grasping stability [13,14].

Many tactile sensor studies have focused on multi-axial force sensing because they are the key information while interacting with objects [7,8,9]. In the case of normal force, grasping force should be controlled depending on the fragility of the objects. Especially, intrinsic properties, like softness, can only be sensed by tactile feedback (instead of visual feedback) so controlling grasp force is necessary for object-adaptive grasping [15]. Sensing shear force is another important factor as it can prevent slip beforehand [16,17]. However, detecting shear force is not always possible in the real world in cases of fast transition from shear force to slip due to sudden impulsive force or a change in the end-effectors’ positions [18]. Therefore, slip-detecting functionality has been reported as a salient factor in tactile sensors, and many works have focused on developing slip-detection functionality [19,20]. In this study, we assert the necessity of tactile sensors to have normal and shear force measuring capabilities alongside slip detectability for stable grasping.

Tactile sensors for robotic applications have been achieved with many sensing methodologies such as piezo-resistive [21,22], piezo-capacitive [12,23], piezo-electric [8,24], magnetic [6,9], optical [17,25], and barometric [4,15,26,27,28,29,30]. Among various types of sensors, barometric sensors have been utilized as tactile sensors due to their several advantages. They are inexpensive, robust, small in size, easy to customize, and simple to use [4,15,26,27,28,29,30]. A barometric pressure sensor chip (BPSC) has an analog-to-digital (A2D) conversion board embedded inside so that it does not require additional circuits or filtering processes. Moreover, they can easily be utilized by fabricating a printed circuit board (PCB) by placing them in desired shapes or positions. Howe et al. adopted BPSC as a tactile sensor by casting soft material on the top to measure normal force [26]. Koiva et al. arrayed multiple BPSCs for localizing contact force, and Clercq et al. detected dynamic events based on localized force [4,27]. By wiring BPSCs with flexible and stretchable wire, Jentoft et al. were able to attach the sensing system to curved surfaces [28]. Guggenheim et al. suggested a three-axis force and torque sensor with eight BPSCs, arraying pairs of them symmetrically [29]. Furthermore, Grover et al. adopted arrayed BPSCs for detecting slips with differently shaped objects with the aid of a learning-based method [30]. The functionality and possibilities of a BPSC as a tactile sensor have been verified in many previous works. However, they have mostly focused on sensing normal directional force due to its sensing principle or require many BPSCs for multi-axial force sensing, which leads to bulky form factors or complex wirings. In addition, sensing dynamic events with a learning-based method is limited to predefined objects or conditions; thus, it would not perform properly when utilized in real-world applications.

The detection of shear force and slip has also been studied in various tactile sensor-related research. Theoretically, shear force occurs before the slip event, so researchers paid attention to detecting shear force as well as incipient slip. One intuitive method is based on an optical sensor type, which traces the displacement of markers on the gel [17,25]. This sensor can precisely detect the movement of objects on the gel surface and measure 3D geometry with high sensitivity. However, this optical-type sensor requires a large volume for embedding the camera inside the sensor, and there is still a problem in decoupling the type of stimuli when complex stimuli are given simultaneously. Another method is to continuously observe the center of pressure (CoP) with arrayed sensors in high resolution [4,6]. If the sensor is able to localize force, it can track the CoP and observe the movement of the contacted objects. This method seems reasonable, but it should cover a large area for arraying multiple sensors and is preferable to tactile skin or large-area-covering applications. Estimation of shear force can also be used to derive friction coefficients alongside the ratio with normal force [16,31]. The slip will be prevented by modeling the static friction. When the estimated coefficient exceeds the static friction coefficient between grasped object and the sensor surface, the slip takes place. Although, this method requires predefined knowledge of the friction coefficient between the objects. In other words, this method is hard to adopt in the real world, as knowing the coefficients from every object is nearly impossible. Furthermore, an approach to model the transition between shear force to translational and rotational slip with a bump-structure tactile sensor has been suggested by Narita et al. [12]. From this study, the transition from shear force to slip can be mathematically modeled by the bump parameters (i.e., height, diameter) and can be utilized for grasping unknown objects by solving the model. However, more than two bumps should be in contact with the object, so it has low spatial resolution and graspable objects are limited. Other studies introduce methods for detecting initial slip by detecting dynamic forces [8,24] but were not able to detect static force. In summary, measuring normal and shear forces (both static and dynamic) is necessary for tactile sensors to detect gradual transition slip, and instantaneous slip should also be detected with different sensing modalities other than detecting shear force variance. 

Based on the previous works, the requirements of our sensor are as follows. (1) Able to measure both normal and shear force for static and dynamic event detection, (2) measure shear force for slip prevention, and (3) detect slip with independent sensing modality in case of an instantaneous slip. Therefore, we propose a BaroTac, which consists of three BPSCs in a small size of 20 mm diameter, with functionalities of measuring three axial forces and detection of slip even with unknown objects. Normal and shear directional forces can be measured with the dome-shaped soft material cast on the top of the arrayed BPSCs. As the soft material plays a key role in transmitting external force to the BPSCs, the type and thickness of the cast soft material should be considered thoroughly. Therefore, we first conduct a parametric analysis of characterizing sensor performance with different types and thicknesses of soft material with a single-cell BPSC-based sensor. Thereafter, we fabricate the arrayed BPSC-based tactile sensor, which is BaroTac, on the basis of the characterization results. Finally, we prove the practical applicability of the sensor by controlling commercial robotic grippers based on sensor feedback.

## 2. Working Principle and Fundamental Characterization

### 2.1. Sensor Structure 

**Figure 1:**
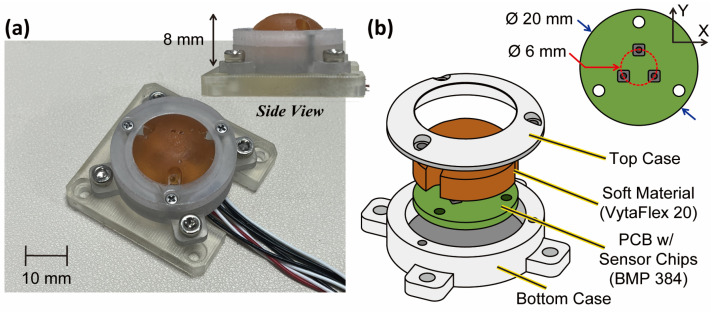
(**a**) Picture of the proposed sensor (asymmetric view and side view). (**b**) Exploded view with dimensions.

The BPSC measures pressure through a sensing hole, and it can be utilized as a tactile sensor with a medium for transferring external stimuli [26]. Generally, soft material is utilized for the medium in the following points. (1) It can transmit stimuli to the sensing hole, (2) protect the sensor from external impact, and (3) provide sufficient friction when the robotic end-effectors contact the objects. Therefore, we cast soft material on top in the thickness of 8 mm, where the bottom 5 mm is cast in a cylindrical shape and the upper 3 mm is in a dome shape (see Figure 1a). In addition, the outer cases (top and bottom) in Figure 1b play two roles in our BaroTac: it forestalls external stimuli coming from unexpected directions and protects the soft material from easily being contaminated. 

A single-cell BPSC-based sensor can sensitively react to external stimuli but is not able to decouple the directions of force due to its sensing principle. Therefore, we array BPSCs in three-phase on the PCB for three-axis force measurement, as shown in the top right of Figure 1b. The cast soft material deforms according to the type of external force and transfers the stimuli to each BPSC. The details of measuring three axial forces are explained in Section 2.3. As the soft material is cast in a dome shape, the peak of the soft material makes ideal point contact regardless of the object’s shape. To elaborate on the preparation process of the BaroTac, the fabrication process is shown in Figure S1.

### 2.2. Characteristics of BPSC-Based Tactile Sensor

#### 2.2.1. Fundamental Characterization

**Figure 2:**
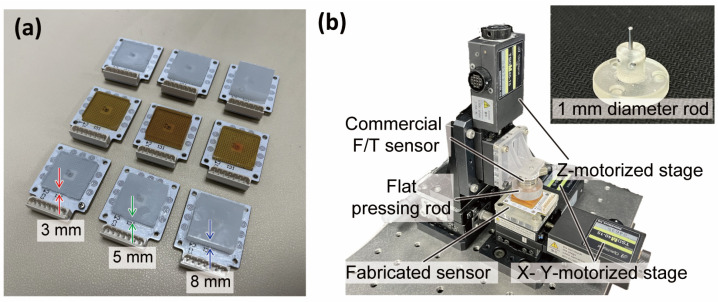
(**a**) Single BPSC-based sensors with different soft materials. (row) Different material types. (column) Different thicknesses. (**b**) Experimental bench for fundamental characterization with three motorized stages, reference sensor, and two types of rods.

The soft material plays an important role in transmitting external stimuli and should be carefully considered to meet the requirements of the sensor specification. Therefore, we investigate the fundamental characteristics of single-cell BPSC-based sensors with different types of soft materials and thicknesses to set the sensor design parameters. Shown in Figure 2a, a total of nine samples are taken into consideration with different thicknesses (3, 5, and 8 mm) and different shore hardness (EcoFlex 00-20 (0.2), VytaFlex 20 (20A), and MoldStar 31T (30A), Smooth-On Inc., Dallas, TX, USA). For each sample, soft material is cast 20 × 20 mm on the PCB while the BPSC (BMP384, Bosch, Germany) is located at the center. 

To characterize each sample to discover the maximum measurable force, linearity, and hysteresis ratio between the loading and unloading, an experimental bench is organized, as in Figure 2b. The Z-motorized stage presses the sensor with a commercial F/T sensor (Nano 17, ATI Industrial Automation, Apex, NC, USA), and X- Y-motorized stages move the sensor in the two-dimensional plane. A flat pressing rod presses the sensor until the single BPSC-based sensor reaches the maximum value.

**Figure 3:**
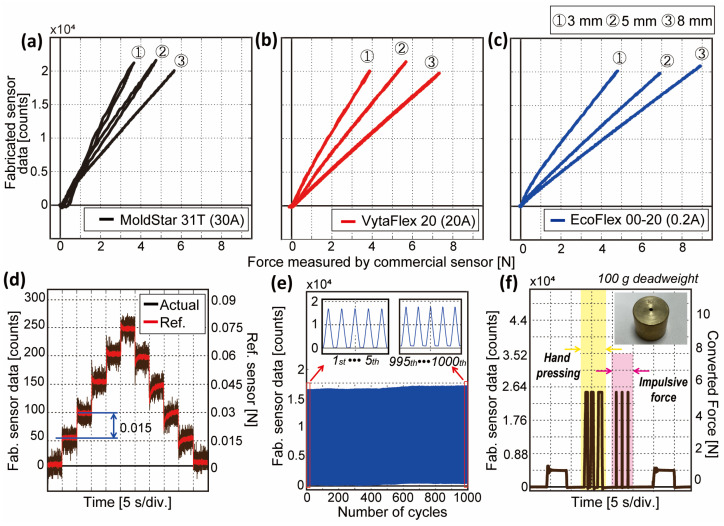
Experimental results of fundamental characteristics with different cast soft materials and thicknesses. (**a**–**c**) Experimental results of MoldStar 31T, VytaFlex 20, and EcoFlex 00-20, respectively, in force (N) to sensor data (counts). (**d**) Sensitivity for VytaFlex 8 mm. (**e**) Repeatability of VytaFlex 8 mm. (**f**) Robustness of single BPSC-based tactile sensor with excessive stimuli.

The result of the characterization is shown in Figure 3a–c, while specific results are tabulated in Table 1. A sensor with thick cast soft material (i.e., 8 mm) shows higher measured force capacity while thin soft material (i.e., 3 mm) shows vice versa for all three materials. Moreover, soft material with a smaller shore hardness (i.e., EcoFlex) is measured to have a bigger force capacity since the material absorbs external stimuli with a big damping effect. With more shore hardness (i.e., MoldStar), it shows less linearity with a considerable hysteresis ratio (see Table 1). Therefore, EcoFlex and VytaFlex were taken into consideration as the types of soft materials for the proposed sensor. Further experiments of the given samples regarding hysteresis ratio under differing contact velocities and amplitudes of pressure are shown in Figures S2 and S3, respectively. In brief, faster velocity and higher pressure lead to the occurrence of a large hysteresis ratio and contacting conditions highly affect the sensor performance.

In order to verify the high sensitivity, repeatability, and robustness of the BPSC-based sensor, which are the known advantages of the BPSC, we conducted additional experiments. We pressed the sensor with the least detectable force for five steps and moved back. Figure 3d shows the sensing resolution of 0.015 N in the VytaFlex 20 8 mm sample, which is estimated as 493-fold of the maximum force (7.396 N). The force resolution results of the rest of the samples are shown in Figure S4. Figure 3e shows the repeatability over 1000 cycles with the VytaFlex 8 mm sample. The flat rod presses the sample in 1 mm/s with maximum force and unloads to zero. After loading more than 500 cycles, the sensor data are slightly saturated, and the difference between the first and last five cycles is calculated to be 0.1%. From this result, we can assert that the sensor is reliable over 500 cycles and even can be utilized for 1000 cycles with low error. Further experiment results showing dynamic response and recovery time from the nine samples are shown in Figure S5. The bigger the shore hardness is, the bigger the restoring power it has. Therefore, EcoFlex and VytaFlex require a recovery time of fewer than 0.2 s, while MoldStar requires more than 1 s to be fully restored. The detailed explanations are included in the caption in the figure.

For the robustness test, the sample is fixed on the flat plane, and a deadweight of 100 g is placed on the sensor surface. After removing the deadweight, the person presses the sensor sample hard and applies impulsive force with a rubber mallet three times until the sensor data exceeds the maximum value. After 5 s of the resting phase, the deadweight is placed again on the sensor surface. The result is shown in Figure 3e, where the hand-pressing part is colored yellow, and the impulsive force part is colored red. When the sensor value is converted to the force on the basis of the results from Figure 3b, it successfully estimates the weight of the deadweight (100 g ≈ 0.98 N) even after pressing or tapping hard. The sensitivity and robustness of the sensor are shown in Video S1. 

#### 2.2.2. Investigation of Sensible Zone

**Figure 4:**
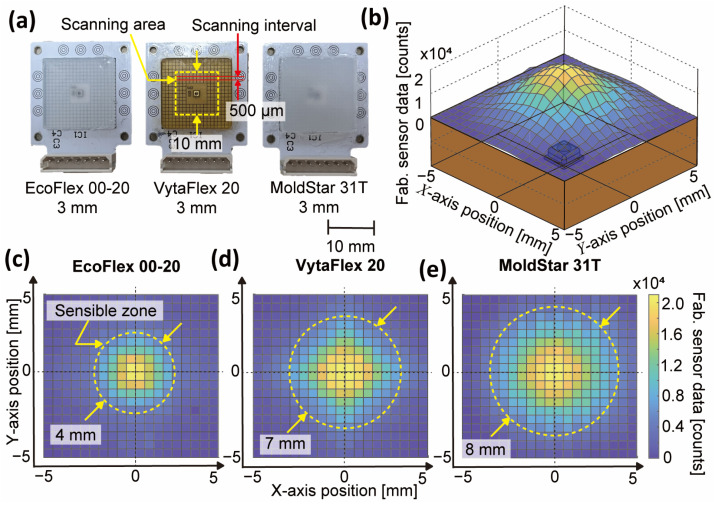
Experimental results of investigating sensible zone. (**a**) Picture of samples with scanning area and interval. (**b**) Sensing result in 3D of VytaFlex sample, showing sensing point close to the sensing hole reacts sensitively. (**c**−**e**) Sensing results in 2D where bigger shore hardness material has a wider sensible zone.

To measure the three-axis force with the BPSC, which is a mono-axial pressure sensor, placing multiple BPSCs in an array is one of the solutions. This section shows the analytic results of sensible zone size to decide the type of soft material and the distance between the three BPSCs when arraying them in three-phase. Among the nine samples shown in Figure 2a, three samples with a thickness of 3 mm (first column in Figure 2a) have been chosen, as thicker material would transfer a wider range of stimuli. Under the same experimental bench in Figure 2b, a 1 mm diameter rod is placed under the reference sensor and presses the 10 × 10 mm area on the sensor surface in the scanning interval of 500 µm. The pressing depth is set equivalent at all 400 scanning points, which is set to be the depth of the sensor value reaching the maximum count at the center point. Figure 4b schematically shows the scanning result of the sample of VytaFlex 3 mm in 3 dimensions. It responds sensitively at the center point near the BPSC while showing less sensitivity far from the BPSC.

Figure 4c–e presents the results of the sensible zone of EcoFlex, VytaFlex, and MoldStar 3 mm samples, respectively. Soft material with small shore hardness (i.e., EcoFlex) shows a small sensible zone size of Ø4 mm as the material absorbs the external stimuli. The large shore hardness sample (i.e., MoldStar) has a zone size of Ø8 mm and has the biggest range due to the material property of less damping. For our sensor, a bigger sensible zone is desired to minimize dead zone and to effectively measure three-axis force by placing the BPSCs wider. Hence, the materials with bigger shore hardness were the candidates. However, according to the fundamental characteristics results from Figure 3a and Table 1, MoldStar has the smallest maximum force and linearity with big hysteresis. In other words, there is a trade-off relationship between force capacity, linearity, hysteresis, and sensible zone size. A compromise for the proposed sensor is VytaFlex, and the thickness is set to be 8 mm to meet the larger force capacity requirement.

### 2.3. Sensor Calibration

**Figure 5:**
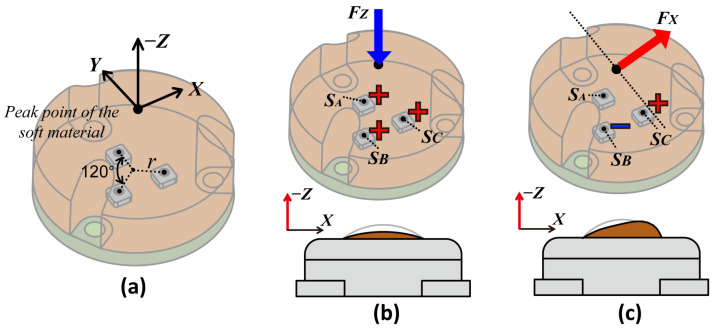
Schematics of measuring three-axis force with coordinate system. (**a**) Isometric view of soft material and three-phase-arranged BPSCs. (**b**) Data increasement with $F_{Z}$ and (**c**) data variance with $F_{X}$ from three BSPCs.

This section shows the calibration method for deriving a three-axis force from BaroTac. The combination of reactivity upon three BPSCs can theoretically discriminate the type of three axial forces, as many researchers have made approaches [32,33]. From the high linearity characteristics derived from Section 2.2.1, linear mapping can be established between the sensor data and the three-axis force. Figure 5a–c schematically depicts the arrangement of the three BPSCs, and the deformation of the soft material in accordance with the force type. Ideally, normal force ($F_{Z}$*)* increases the sensor data of all BPSCs in the same ratio, while shear force in the X-direction ($F_{X}$) increases the sensor data from $S_{C}$, decreases the data in $S_{B}$, and causes no change in $S_{A}$. 

The three-axis force can be derived with the calibration matrix and three sensor data ($S_{A},\;S_{B},\;S_{C})$ in accordance with the geometrical relationships. This can be expressed as:

(1) $$\left[\begin{array}{c} \;F_{X}\;\\ F_{Y}\\ F_{Z} \end{array}\right]=\left[\begin{array}{ccc} 0 & \beta rsin\left(\pi /3\right) & -\gamma rsin\left(\pi /3\right)\\ \alpha r & -\beta rcos\left(\pi /3\right) & -\gamma rcos\left(\pi /3\right)\\ k & k & k \end{array}\right]\;\left[\begin{array}{c} S_{A}\\ S_{B}\\ S_{C} \end{array}\right]=:C\;\left[\begin{array}{c} S_{A}\\ S_{B}\\ S_{C} \end{array}\right]$$

where α,β, γ are converting coefficients from $S_{A},\;S_{B},\;S_{C}$ having the unit of N/mm, k is the ratio coefficient having the unit of N, and r denotes the radius from the center (3 mm). For more details of mathematical analysis with three-phased-arranged sensors, refer to [32]. 

Equation (1) ideally works for solid material where torque happens on the rigid body. Therefore, we need an additional experiment to derive the calibration matrix, C, to verify the sensor response tendencies (i.e., magnitude and sign of the coefficient value). In the experimental bench shown in Figure 2b with the flat pressing rod, three sensor data $S_{A},\;S_{B},\;S_{C}$ are obtained, and three-axis force data $F_{X},\;F_{Y},\;F_{Z}\;$are obtained from the reference F/T sensor. C is derived by a pseudo-inverse matrix as:

(2) $$C={\left[\begin{array}{c} F_{X\;}\;F_{Y\;\;}F_{Z} \end{array}\right]}^{T}{\left({\left[\begin{array}{c} S_{A}\;\;S_{B}\;\;S_{C} \end{array}\right]}^{T}\right)}^{-1}$$

(3) $$∴C=\left[\begin{array}{ccc} -3.41 & 3.77 & -3.72\\ 4.77 & -2.06 & -2.16\\ 3.03 & 3.39 & 4.53 \end{array}\right]\times 10^{-5}.$$

From Equation (3), the tendencies from each sensor match chiefly. However, there are some noticeable differences (e.g., (1, 1) component, magnitude difference in (3, 1) to (3, 3)), and the predictable causes for this are as follows. (1) Different sensitivity between the sensors, (2) not perfectly aligned calibration process, and (3) hand-fabricated. With the obtained calibration matrix, the normal force measurement capacity of BaroTac is around 5.5 N, and the shear maximum force is around ±3.3 N.

### 2.4. Slip Detection Method

The slip detection of the tactile sensor can be achieved with diverse methods (i.e., friction coefficient, displacement, vibration, etc.), as many previous works have suggested [19]. Ideally, the slip occurs after the shear force and is apt to cause high fluctuations in the sensor signal when sufficient friction is guaranteed between the grasped object and the sensor surface. However, shear force before slip cannot always be observed in many cases in real-world applications due to technical limitations. When it comes to human hands, they detect skin stretch before the slip, and after the slip takes place, the skin detects a vibration from the mechanoreceptors and immediately reacts to strengthen the grasp force so as not to drop the object [34]. Thus, we tried to detect vibration caused by the slip event with another sensing modality (in addition to measuring shear force), which is to analyze frequency. With this approach, the slip can be detected with the property-unknown objects and does not require any learning-based methods. In other words, this method can be utilized in an unstructured environment. 

For the frequency analyzing method, the Fourier transform (FT) is one of the well-known methods in slip detection tactile sensors in that it decomposes the signal into sin and cosine waves to obtain frequency information [35]. However, FT-based methods require a fixed-size time window to be analyzed in real-time. The length of the time window has a trade-off between fast adaptive real-time analysis and loss of information [36,37]. Another method is discrete wavelet transform (DWT), which disassembles the signal into high and low frequencies in the desired frequency range based on the sampling rate [38]. The DWT decomposes the signal in different levels with high and low pass filters, and the DWT result is indicated as wavelet coefficients. We deal with high-pass filtered signals in each level, and each contains different frequency ranges. At the higher level, it contains a lower frequency range. For instance, with a sampling rate of 100 Hz, the 1st level contains a 50–100 Hz signal, the 2nd level contains 25–50 Hz, and so on in the same manner. In our case, we need high-frequency filtered data to discriminate slip from other stimuli, and thus we need lower levels (e.g., 1st or 2nd level). Because DWT can analyze frequencies in the time domain, it has been regarded as a promising method for real-time sensor applications [39,40]. In this study, we adopted 2nd level Haar DWT for slip detection [41], and further details are explained in Section 3.2. Moreover, setting a threshold is necessary for decoupling slip, so we set the threshold to be three times the standard deviation of the noise on the basis of the three-sigma rule. With this threshold, our sensor is able to decouple slip from other stimuli and properly detect slip with common objects.

**Table 1:** Characteristics of BPSC-based sensors according to the type of cast soft material with different shore hardness (0.2, 20, and 30 A) and thicknesses (3, 5, and 8 mm).

Characteristics	EcoFlex 00-20 [42]			VytaFlex 20 [43]			MoldStar 31T [44]			
Shore Hardness	0.2			20 A			30 A			
Thickness [mm]	3	5	8	3	5	8	3	5	8	
Force Measurable Capacity [N]	4.801	6.998	8.894	3.915	5.699	7.396	3.662	4.763	5.636	
Hysteresis ratio (%)	2.386	1.933	1.081	2.678	1.447	1.066	7.656	5.98	2.996	
Linearity	0.997	0.998	0.999	0.999	0.999	0.999	0.973	0.987	0.999	

## 3. Experimental Validation

On the basis of the design parameters, we fabricate BaroTac and suggest three-axis force measuring and slip detection methods from the previous section. This section verifies the proposed sensor’s performance and functionality by discussing experimental results. Furthermore, we present the sensor’s capability to detect slip using DWT apart from the normal and shear forces. 

### 3.1. Three-Axis Force Measurement Capability

**Figure 6:**
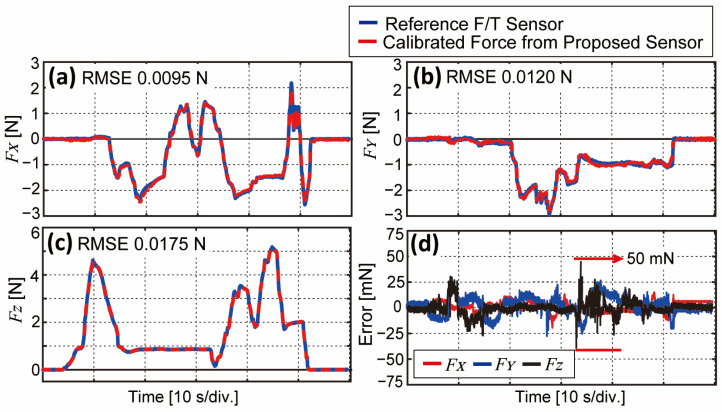
(**a**–**c**) Results of the calibrated forces in X-, Y-, and Z-axis, respectively, with RMSE. Blue solid lines indicate reference force, and red dashed lines indicate calibrated force. (**d**) Force error in three-axis, which is smaller than 50 mN in all three axes.

From the calibration matrix obtained from the previous step, we prove its sensing accuracy with the same experiment bench shown in Figure 2b. While the calibration matrix is obtained with motorized stages moving automatically, the verification experiment is conducted with random cases. A person moves the three stages manually with random forces and directions for 60 s, and the calibrated force is compared with the reference F/T sensor. The compared result is shown in Figure 6 with forces in the three-axis, root mean square error (RMSE) value, and force error. Figure 6a,b show the results of X- and Y-directional calibrated force with high compliance and linearity. The maximum shear force measurement capacity is observed as ±3.3 N, and RMSE is around 0.01 N. Figure 6c shows the calibrated normal force also with high compliance and linearity, and the force capacity in the Z-direction is around 5.5 N. Figure 6d is the force error in all three-axis, where slightly big error is observed between 30 to 40 s in Z-directional force (black line) where the loaded force suddenly decreases and increases again. This is possibly due to the property of soft material in that it requires a certain amount of time to be restored to its original state, referring to the Figure S5 results. However, when the stimulating phase is completely over (after 50 s), all sensors are set to zero. The results prove that calibration matrix C can be applied to our sensor data to measure three-axis forces with a mean error of below ±50 mN. The proportion between the RMSE and force measurement capacity from each axis indicates the error in percentage, which is calculated to be 0.18, 0.14, and 0.3% in the X-, Y- and Z-axis, respectively. In addition, Table 2 shows the comparison of the force measurement capacity of the previous three-axis force tactile sensors with the dimensions and sampling rate.

### 3.2. Slip Detecting Capability

In this study, we adopted DWT for our slip detection method, which is an efficient method of extracting high-frequency components in a time domain. DWT is a suitable method for detecting dynamic events, and these dynamic events occur when contacting or releasing an object, as well as slipping. The difference between these two types of events is that whereas contacting or releasing phases contain low-frequency signals, slip contains high-frequency signals. As aforementioned, DWT analyzes signals in different frequency ranges depending on the levels; it is necessary to utilize the proper level for slip detection and decoupling from other external stimuli (i.e., normal or shear forces). 

**Figure 7:**
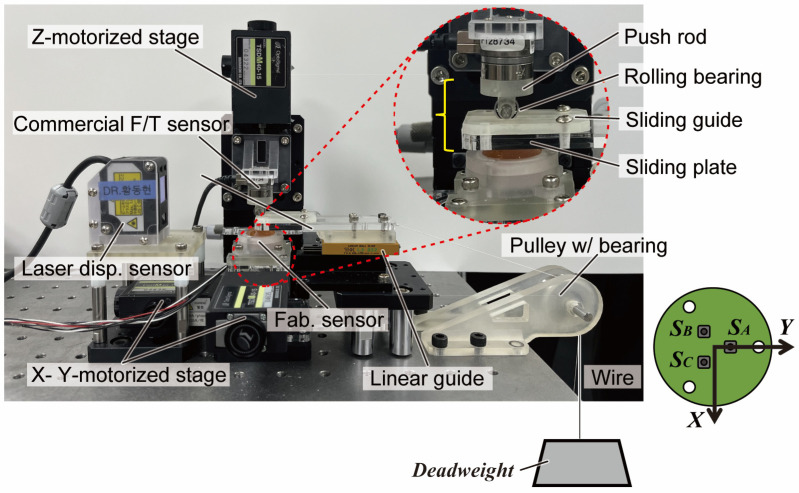
Experimental bench for determining normal, shear force, and slip decoupling capability with three different deadweights (50, 100, and 200 g) causing slip. Rolling bearing installed at F/T sensor minimizes frictional effect as in the close-up picture.

To validate two main factors for detecting slip from DWT, which are (1) slip detectable level of DWT and (2) decoupling ability from normal and shear forces, we compose another experimental bench with deadweights, as shown in Figure 7. In addition to the previous bench, a laser displacement sensor (ZX2-LD50, OMRON, Kyoto, Japan) works as another reference sensor for slip detection ground truth. In order to minimize the frictional effect of the push rod while slipping, a rolling bearing is installed at the commercial F/T sensor, as can be seen in the close-up picture in Figure 7. The bearing rolls down the sliding guide with a rail when a slip happens. An acrylic sliding plate is in contact with the sensor surface, sliding along the linear guide as deadweight pulls the plate in the Y-direction. Here, three different deadweights (50, 100, and 200 g) are hung on the wire. For the experimental protocol, we conduct two different experiments with this bench, which are (1) level of DWT, which properly decouples slip, and (2) decoupling ability of shear force and slip.

**Figure 8:**
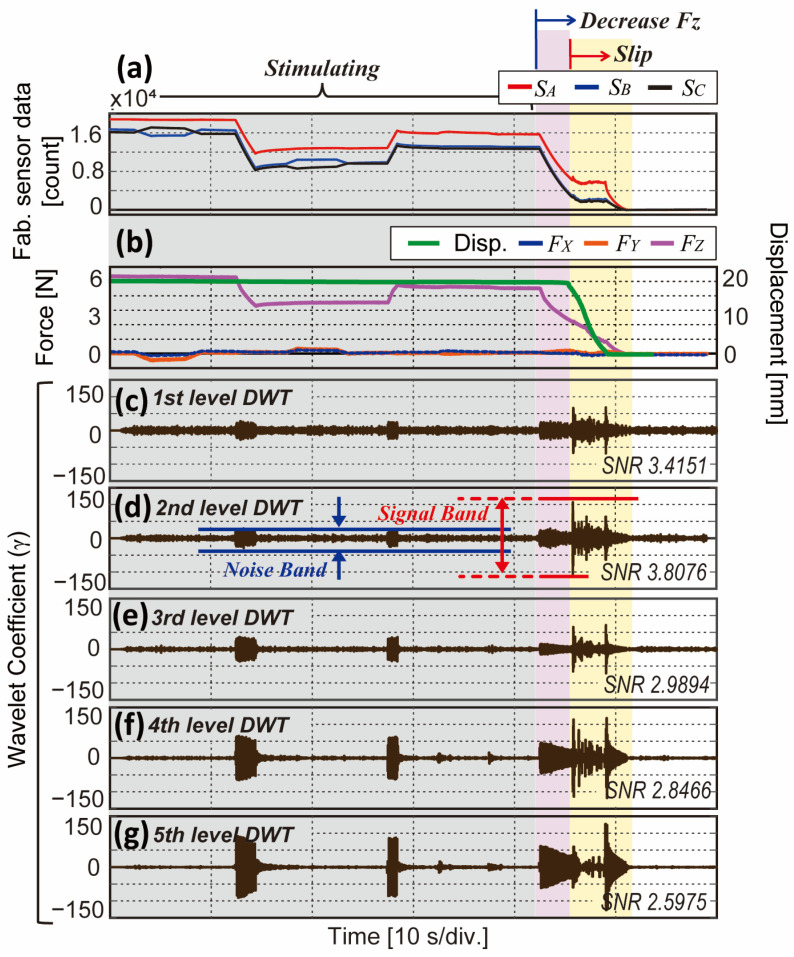
Experimental results of slip detection in different DWT levels. Red area indicates stimulating period with random three-axis forces and yellow area indicates slip. (**a**) Fabricated sensor data from each BPSC. (**b**) Three-axis forces and displacement from reference sensor. (**c**–**g**) DWT results in the 1st to 5th level with SNR value.

In our first experiment, the reference F/T sensor with a Z-motorized stage presses the sensor with the maximum force of the proposed sensor, and 100 g deadweight is hung at the end of the wire. While the X- and Y-motorized stages move the sensor in a shear direction randomly, the Z-motorized stage decreases or increases its normal force simultaneously. After stimulating the sensor, the Z-stage moves up slowly and releases the sliding plate to cause a slip under the effect of the deadweight. The moment of slip can be referenced by the displacement sensor. We observe DWT in the 1st to 5th level to decide which level detects slip with the most evident peak. This distinctiveness can be quantitively expressed with a signal-to-noise ratio (SNR) in a logarithmic scale as follows:

(4) $$\mathrm{SNR}=\log\frac{\gamma_{\left(signal\;band\right),\;P-P}}{\gamma_{\left(noise\;band\right),\;RMS}}\;$$

where γ denotes the wavelet coefficient from the DWT. A denominator for the noise is calculated as the root mean square (*RMS*) value of the non-slip band (blue lines in Figure 8d), and a numerator for the signal is calculated as the peak-to-peak value of the slip band (red lines in Figure 8d). Higher SNR implies that there are prominent peaks during the slip.

Figure 8a,b show the experimental results of raw sensor data from the three sensors and reference sensor data. The sensor is stimulated with random normal and shear forces until 42 s, and after, normal force starts to decrease, and slip initiates at 45 s. From Figure 8c–g, DWT results (i.e., wavelet coefficients (γ)) in the 1st to 5th level are plotted, respectively. In Figure 8c,d, peaks protruded with slip occurrence compared to other stimuli, while Figure 8e–g also show big γ during the normal and shear force stimulating phase. This implies that the slip contains a wide range of frequencies while normal and shear forces contain a relatively lower range of frequencies. The SNR in each level represents the result with numerical indexes. We adopted 2nd level DWT in this study as it shows higher SNR than the 1st level. Considering the sampling frequency of our BaroTac (300 Hz), 2nd level DWT contains a frequency range of 75 to 150 Hz that our proposed sensor can detect high-frequency from the slip event [19]. 

Our second experiment has almost the same protocol as the previous experiment. The Z-motorized stage first presses the sensor at maximum normal force (5.5 N) and slowly releases the sliding plate after a few seconds. Following is the shear force, and the slip takes place subsequently. The $F_{Z}$ from the F/T sensor and displacement from the laser sensor are referenced, the 2nd level DWT is analyzed, and the three-axis force is measured on the basis of raw sensor data with the calibration matrix obtained in Equation (3).

**Figure 9:**
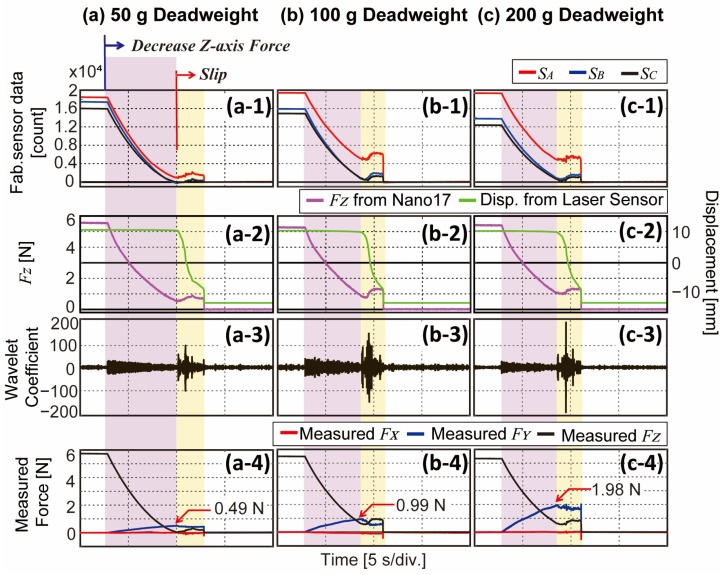
Experimental results of decoupling slip from Y-axis force. (**a**–**c**) Results with different deadweights (50, 100, 200 g). (**a1**,**b1**,**c1**) Fabricated sensor data from each BPSC. (**a2**,**b2**,**c2**) Z-axis force and displacement from reference sensors. (**a3**,**b3**,**c3**) DWT results in the time domain. (**a4**,**b4**,**c4**) Measured force calculated from fabricated sensor data. Measured $F_{Y}$ indicates weight of deadweight.

The result is shown in Figure 9 with three different deadweights. Figure 9(a1,b1,c1) indicates the sensor output from the three sensors. As the experiment is recorded after the deadweight is hung, there exists a difference in sensor data between $S_{A}$ (red line) and other sensors (blue and black lines) at the starting point, which indicates shear force is being applied to the sensor. Note that the bigger difference is shown in Figure 9(c1) than in Figure 9(a1) as heavier deadweight is hung to cause a bigger shear force. We can also discover from the raw sensor data that the slip area (red area) has noticeable fluctuations compared to the other area. Starting around 2.5 s, normal force starts to decrease and around 10 s, slip initiates. This can be noticed from Figure 9(a2,b2,c2) that the $F_{Z}$ decreases and displacement starts to decrease at 10 s. Note that the maximum measurement range of the displacement sensor is around 22 mm. Figure 9(a3,b3,c3) are the 2nd level DWT results which show high peaks as slip occurs. It can also be noticed that with heavier deadweight causing slip, bigger peaks exist in the DWT result. The last row (Figure 9(a4,b4,c4)) shows measured force from the sensor raw data in Figure 9(a1,b1,c1). Regarding the experimental bench shown in Figure 7, the slip occurs in the Y-direction, and the measured $F_{Y}$ (blue line) increases until it reaches the force of the corresponding deadweight (i.e., 50 g = 0.49 N, 100 g = 0.98 N, 200 g = 1.96 N). The measured force in the Z-axis matches the trend of the $F_{Z}$ from the reference sensor (purple line). From this experiment, while three-axis forces are being accurately measured, the slip can be detected with DWT apart from the shear force.

## 4. Demonstration of Practical Applicability

**Figure 10:**
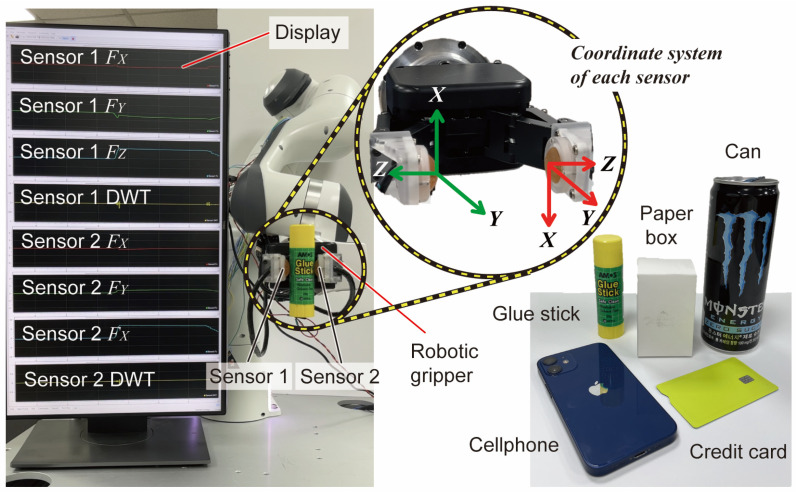
Experimental setup for practical applicability with common objects. Real-time plotting on the display of two sensors including three-axis forces and DWT result. Coordinate system of each sensor when installed on robotic gripper.

In order to verify our proposed BaroTac, we show its practical applicability with commercial robotic grippers in this section. A stable grasp can be ensured if the gripper can move under the given tactile feedback. We conduct two types of experiments, which are (1) to increase weight and decrease grasp force and (2) to grasp everyday objects with external interference force. The experimental features are presented in Figure 10. Two fabricated sensors are installed at the tip of a commercial parallel robotic gripper (RH-P12-RN, ROBOTIS Co., Seoul, South Korea) with a grasping stroke of around 100 mm. The display shows each sensor data, measured three-axis force, and DWT results in real time. As each sensor is calibrated in the same manner, the coordinate system matches, and the installed position of the sensor should be considered (see close-up picture in Figure 10). Figure 10 also includes the target objects with different shapes, surface roughness, and weights.

### 4.1. Verification of Sensing Performance on Robotic Gripper

The first type of experiment has two sessions which are to increase and decrease the weight of the grasped object. For the case of increasing weight, the gripper grasps the empty cup with sufficient force and waits for 5 s. Thereafter, a person adds a liquid of 500 g into the cup. If the measured shear force in the X-direction exceeds the set threshold from either sensor, the gripper strengthens the grasp force. To check whether the cup is stably grasped, the person shakes the cup with a big enough force (see Video S2 for more details). In this experiment, we command the gripper to strengthen grasp force only once when either of the sensors first detects slip based on the DWT results. 

**Figure 11:**
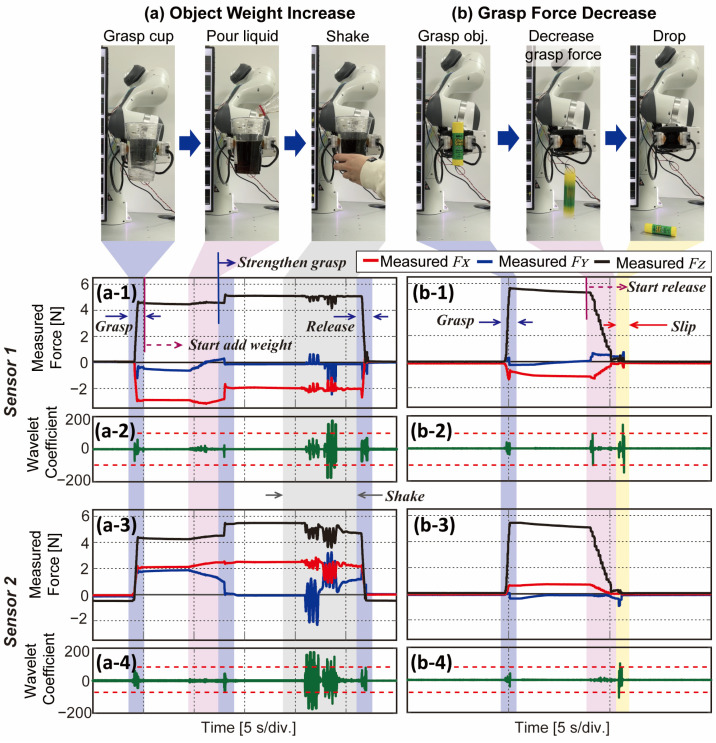
Experiment process and results of (**a**) increasing weight and (**b**) decreasing grasp force. (**a1**,**b1**) Three-axis force measured from sensor 1. (**a3**,**b3**) Three-axis force measured from sensor 2. (**a2**,**b2**) DWT results with threshold from sensor 1. (**a4**,**b4**) DWT results with threshold from and sensor 2.

The sequence of this session and corresponding results are shown in Figure 11a. As can be seen in Figure 11(a1,a3), the $F_{X}$ (indicated in red lines) changes after starting to add weight around 10 s (red area). The gripper strengthens grasp force around 13 s as the $F_{Z}$ (black lines) increases in both sensors. From the period when the $F_{Z}$ increase, there is no more change in shear force, which can imply that the cup is stably grasped. The gray area indicates the section where the person grasps the cup and shakes to check the grasped state. Figure 11(a2,a4) show DWT results and set thresholds from sensor 1 and sensor 2, respectively. Except for the shaking phase, coefficients did not exceed the threshold (green lines), which means there was no slip during the pouring phase (Video S2). Because the contact between the sensors and grasped object cannot be perfectly aligned as a person gives the object, the magnitude of $F_{Z}$ may vary in the two sensors. Moreover, the grasped object has attracting force downward by gravity, $F_{X}$ vary in big magnitude in both sensors—negative in sensor 1, positive in sensor 2 (red lines). The amplitude of variance in $F_{Y}$ is smaller than $F_{X}$ but still varies. This is possibly due to the misalignment of the grasped object, and they are set to zero after the grasp force is strengthened (blue lines). Due to the misalignment, the gripper operates when either of the sensors exceeds the threshold. This experiment shows the ability of the sensor to provide the state information of unstably grasped objects by observing shear force in real-time and the gripper to react accordingly. 

In the case of decreasing the grasp force, the gripper grasps the object with maximum force and slowly decreases the grasp force to release the object. The session is depicted in Figure 11b with experimental results. After the gripper starts to decrease its grasp force (red area in Figure 11(b1,b3)), the $F_{Z}$ of each sensor (black line) decreases, and shear forces vary. One interesting phenomenon is that the DWT exceeds the threshold from sensor 1 at around 9 s in Figure 11(b2) in the red area. This is because the grasped object was slightly tilted towards sensor s1 due to lack of grasp force, and sensor 1 detects slip at this moment. Video S3 presents this phenomenon graphically, and refer to it for more details. After a few seconds, both DWT results exceed the threshold as gross slip happens and the gripper drops the object. From this experiment, our proposed sensor can detect slip and observe unexpected happenings from the grasped object in real time.

### 4.2. Feedback Control of Gripper for Stable Grasping

With the objects shown in Figure 10, we conduct an additional experiment on our sensor to detect slip properly when external interfering force is applied. In this experiment, the position of the gripper changes from Figure 10, and thus $F_{Y}$ in both sensors are parallel to the direction of gravity. We predefined initial grasp force as 1 N, and a person pulls an object to the gravity direction, which can act as a sudden interference force. Five common objects with different shapes, surface roughness, and weight (i.e., glue stick, paper box, can, cellphone, and credit card) are selected as target objects. When an external force is applied to the grasped object, the sensors detect slip based on the DWT results and send commands to strengthen the grasp force to the gripper. The command is sent only once in this experiment to strengthen grasp force. After assuming the objects are stably grasped, the person shakes the object to check grasped state. The full-length video is shown in Video S3 with five different objects and a real-time signal display.

**Figure 12:**
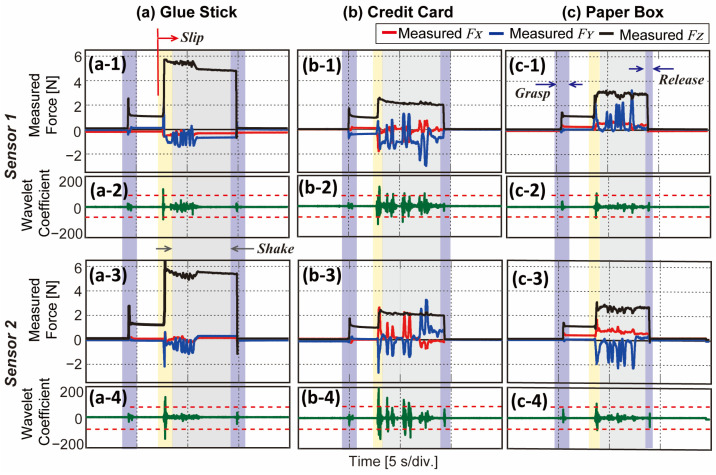
Experimental results of slip detection experiment with external interfering force. (**a**) Results of glue stick grasping. (**b**) Results of credit card grasping. (**c**) Results of paper box grasping. (**a1**,**b1**,**c1**) Three-axis measured force from sensor 1 and (**a3**,**b3**,**c3**) from sensor 2. (**a2**,**b2**,**c2**) DWT results with threshold from sensor 1 and (**a4**,**b4**,**c4**) from sensor 2, respectively.

Shown in Figure 12 are the results with three different objects. Three-axis force from sensor 1 is indicated in Figure 12(a1,b1,a1), and from sensor 2 is indicated in Figure 12(a3,b3,c3). The DWT results from each sensor are plotted in Figure 12(a2,b2,c2) for sensor 1, Figure 12(a4,b4,c4) for sensor 2 (green lines). After grasping the object with a normal force of 1 N (black lines), an external interfering force is applied around 8 s (yellow area). At this moment (8 s), both sensors show significant peaks in DWT results. According to the sensors’ DWT result, the gripper strengthens its grasp force immediately to prevent the object from dropping as the $F_{Z}$ (black line) increases suddenly. It can be noticed that the increased amount of the $F_{Z}$ of inflexible objects (i.e., glue stick, cellphone) is bigger than flexible objects (i.e., paper box, credit card). A person checks the grasped state by shaking and finally releases the object. All objects were stably grasped after strengthening the grasp force, as in Video S3.

## 5. Discussion

Tactile sensing ability in robotic applications can aid robotic end-effectors in performing tasks, especially when interacting with grasped objects. Specifically, multi-axial force and slip detectability are considered salient factors for stable grasping, as many previous works dealt with improving or enhancing these functionalities. In this study, we develop a BPSC-based tactile sensor, which can measure three-axis force alongside the detection of slip. We first characterize the single-cell BPSC-based sensor to identify its fundamental characteristics. As shown in Figure 3, the hardness of the cast soft material and thickness play important roles in sensing performance (e.g., force measurement capacity, linearity, and hysteresis ratio). An additional experiment characterizing a sensible zone to avoid a dead zone is conducted, and the results are shown in Figure 4. From the results in Figure 3 and Figure 4, we notice a certain trade-off relationship with material hardness, sensible zone, and force capacity. Hence, we adopted VytaFlex 20 in 8 mm thickness to the BaroTac. At this point, different types of soft materials or thicknesses can be used to meet desired requirements in other BPSC-based sensors or applications. Based on the characterization results in Figure 3b showing the high linearity, our multi-cell BPSC-based tactile sensor, which is BaroTac, can measure three-axial forces with a calibration matrix shown in Equation (3). The validation process is shown in Figure 6 with RMSE and proportional errors. According to the result, errors in all three axes are lower than ±50 mN, and the error in percentage from RMSE to maximum force is calculated as 0.14, 0.18, and 0.3% in the X-, Y-, and Z-axis, respectively. Considering the force capacity of the BaroTac (5.5 N in the Z-axis, ±3.3 N in the X- and Y-axis), the errors are negligible in that the folds in the three-axis are 323, 550, and 388.

In the next step, we explored its slip detectability with DWT, which is an efficient frequency analyzing method in the time domain. Because the DWT decomposes signals with cascaded filters, each filtered signal contains a different range of frequencies and is plotted in different levels. As the level gets higher, it contains a lower frequency range signal that slip event and other stimuli could not be decoupled. As our aim is to decouple slip with DWT, the first experiment is conducted to find the appropriate level. Figure 8 shows the DWT results in five different levels while other types of stimuli (e.g., normal and shear force) are being applied. Subsequently, we calculate SNR following Equation (4) to evaluate which level shows the most evident peak during slip. According to the result, 2nd level DWT could detect slip aside from normal and shear forces. Meanwhile, a sampling rate highly affects DWT results as higher frequencies can be contained in the signal with a higher sampling frequency. The sampling rate of our BPSC is 300 Hz, which means 2nd level DWT contains a signal in the frequency range of 75–150 Hz. With the sensor having a higher sampling rate, it would be advantageous to obtain slip signals in the higher frequency range. However, our proposed sensor was able to detect slip in 2nd level DWT with a given sampling rate. Figure 9 shows the results of the decoupling ability of slip from the shear force while deadweights are causing the gradual slip. The sensor was able to detect slip as DWT results abruptly increase when slip occurs. It also estimated the magnitude of the shear force (i.e., 1 N for 100 g deadweight) and normal force. 

We finally demonstrate the practical applicability of BaroTac with a commercial robotic gripper. Based on the feedback from our sensor, the gripper reacts to perform a stable grasp of the objects. As in Figure 11a and Figure 12, the gripper reacts in two cases where (1) shear force exceeds the threshold and (2) DWT results exceed the threshold. In the former case, gradual slip can be detected where transmission from shear force to slip happens over a long period of time. The latter case deals with an instantaneous slip that stops incipient slip from becoming gross slip. From the experimental results, the gripper was able to strengthen grasp force with slipping objects having different properties based on our sensor feedback. 

## 6. Conclusions

In this paper, we propose a barometer-based tactile sensor that can measure three-axial force and detect both gradual and instantaneous slips. We conduct an experiment to reveal the fundamental characteristics of BPSC-based sensor performance with different soft material types and thicknesses. On the basis of this result, our proposed sensor is designed and fabricated. Thereafter, we introduce sensing principles and methods and validate them. The three-axial force measurement error in percentage is 0.18, 0.14, and 0.3% in each axis, and it could successfully decouple slip with 2nd level Haar DWT. To verify its functionality in the real world, two BaroTacs were installed at the robotic gripper and controlled the gripper based on the sensor feedback. In both cases of gradual slip and instantaneous slip, the gripper was able to strengthen grasp force and achieve a stably grasped state. 

Our future work will focus on enhancing the sensor performance, including normal force measurement capacity and linearity. To grasp heavier objects stably for wide applications, it is required to have a bigger normal force capacity. Moreover, as we cast soft material on top, low resilience and damping effect are inevitable, and sudden changes in the force will be measured less accurately (see Figure 6d). One of the solutions can be adding a rigid plate that can have flexible motions (e.g., flexure) with the soft material alongside. This adaptation will also enable utilizing Equation (1) as the torque occurs and can mathematically be analyzed with precise measurement. In addition, since adding a rigid plate would affect force measurement capacity, it would be possible to increase the force capacity through structural changes. Another future work is to define the threshold-setting method. Until now, we predefined the DWT threshold base on the noise band, as mentioned in Section 2.4. This set threshold was able to detect the slip of common objects except for some objects with smooth surface roughness. One of the methods is to set the threshold at the initial step when the sensor first comes into contact with the object. It may require a certain algorithm or strategy, and we are planning to work on it in the future. 

## Figures and Tables

**Table 2:** Comparison of force measurement capacity of three-axis force tactile sensors.

Characteristics	Number of Sensor Units	Dimensions [mm]	Force Measurement Capability [N]		Sampling Rate [Hz]
			$F_{Z}$	$F_{X}\;F_{Y}$	
P. Yu et al. [8]	4	8 × 8 × 2.6	1.5	0.5	>400
Y. Wang et al. [40]	4	12 × 12 × 1.4	15	0.6	-
H. Choi et al. [32]	3	Ø40 × 10	13	±2.5	8
D. Vogt et al. [33]	3	Ø10 × 3.5	1.2	±0.45	100
Proposed	3	Ø20 × 8	5.5	±3.3	300

## Data Availability

No new data were created or analyzed in this study. Data sharing is not applicable to this article.

## Supplementary Materials

The following supporting information can be downloaded at: https://www.mdpi.com/article/10.3390/s23010428/s1. Figure S1:Fabrication process of BaroTac; Figure S2: Hysteresis ratio of nine samples under different velocities; Figure S3: Hysteresis ratio of nine samples under different amplitudes of preload; Figure S4: Force resolution of nine samples; Figure S5: Dynamic response of 8 mm thickness samples; Video S1: Sensing performance of BaroTac in real-time; Video S2: Verification of sensing performance: increasing weight, decreasing grasp force; Video S3: Feedback control of robotic gripper for stable grasping.
