# Supplementary material for: BaroTac: Barometric Three-Axis Tactile Sensor with Slip Detection Capability
Source: Sensors (Basel). 2022 Dec 30;23(1):428. doi: 10.3390/s23010428 (PMC9823802; doi:10.3390/s23010428)
Supplement: Supplementary file 1 [file sensors-23-00428-s001.zip › sensors-2095887-supplementary.pdf]

Supporting Information

BaroTac: Barometric Three-axis Tactile Sensor with Slip Detection Capability

Gyuwon Kim and Donghyun Hwang

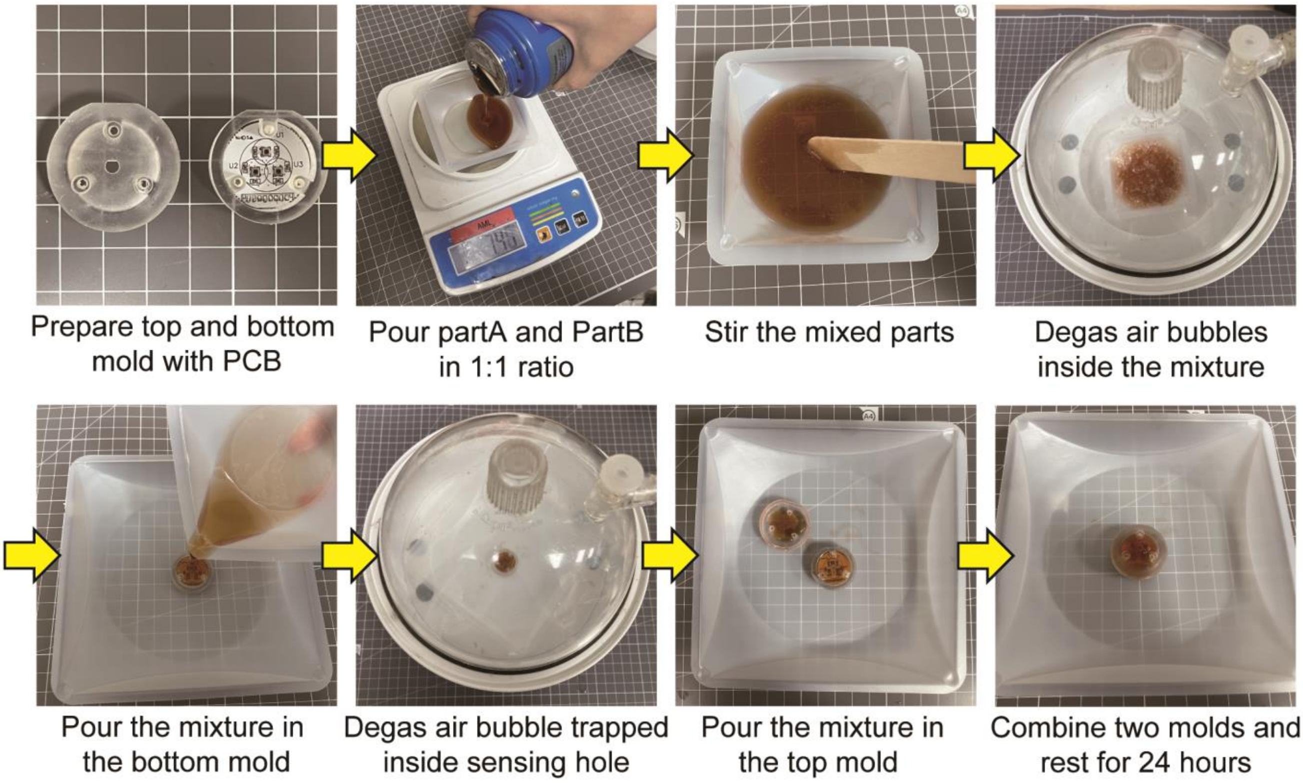

Supplementary Figure S1. Fabrication process of BaroTac. Top mold is dome-shaped and bottom mold is cylindrical shape. Two molds are combined after removing the air bubble trapped inside the sensing hole of BPSC.

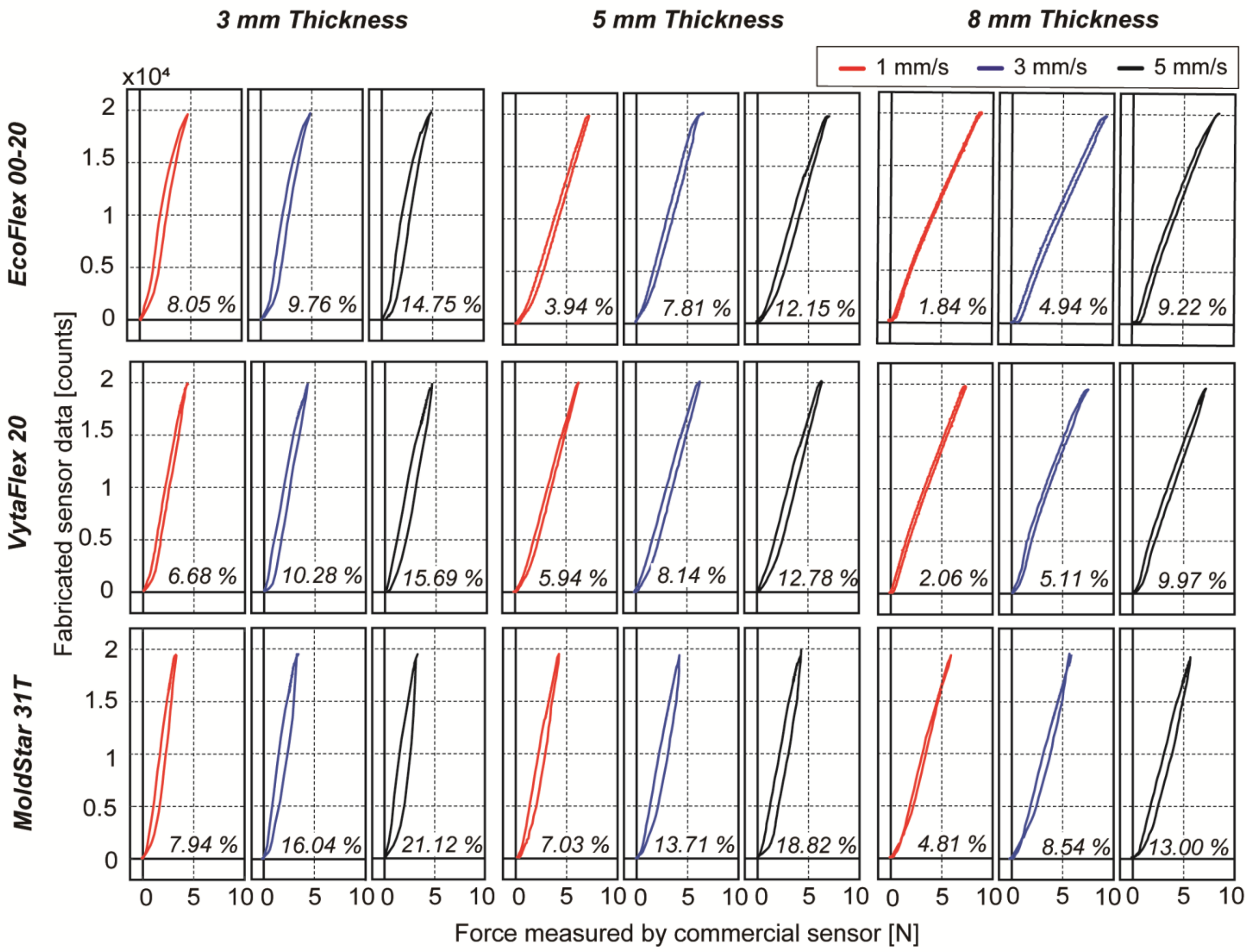

Supplementary Figure S2. Hysteresis between loading and unloading phases under different velocities (1, 3, and 5 mm/s). (a-1) to (a-3) Results of EcoFlex samples with different thicknesses. Flat rod presses the samples with maximum force measurement capacity. (b-1) to (b-3) Results of VytaFlex samples with different thicknesses. (c-1) to (c-3) Results of MoldStar samples with different thicknesses. For all the samples, a bigger hysteresis ratio is shown with 1) faster loading and unloading velocity and 2) bigger shore hardness samples.

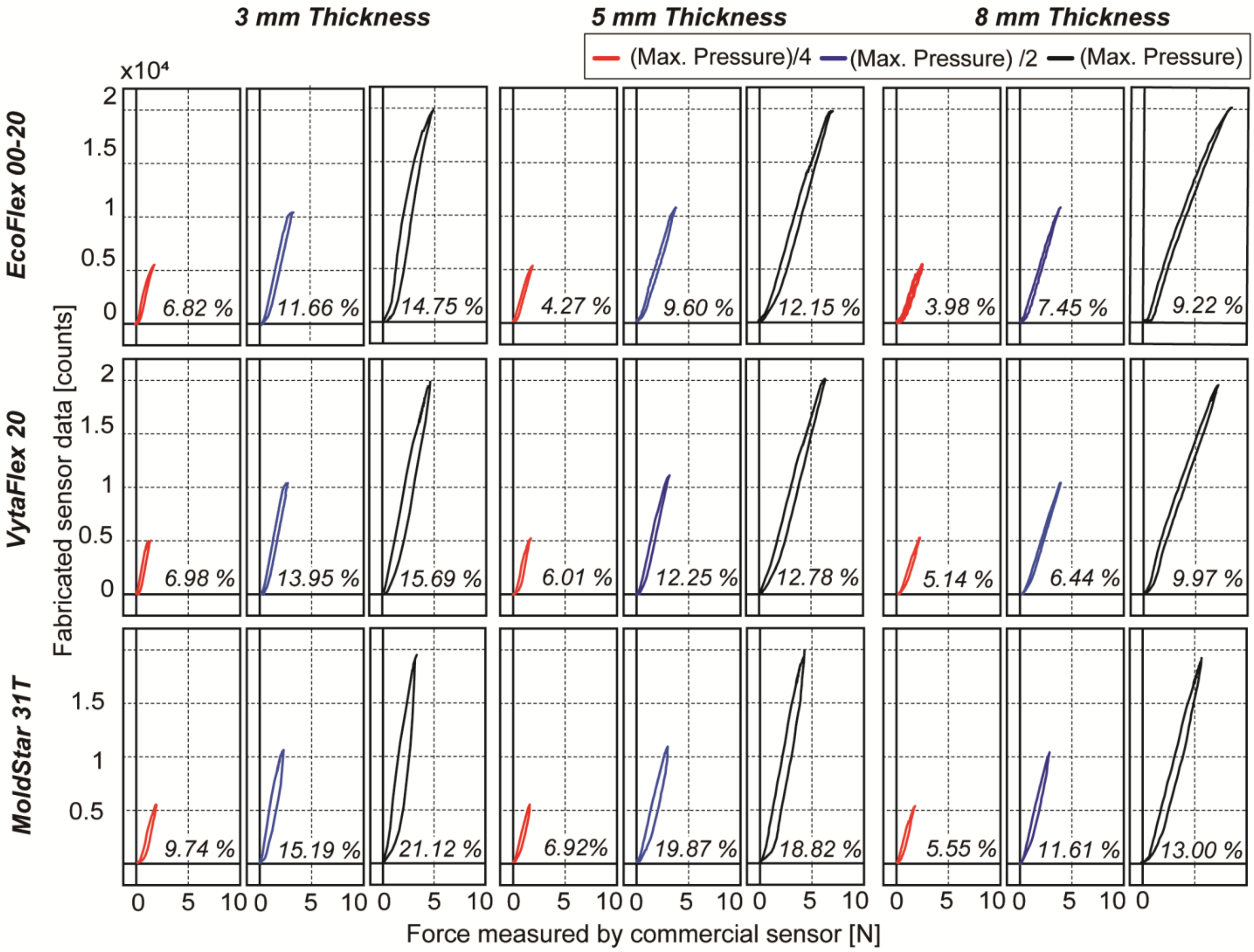

Supplementary Figure S3. Hysteresis between loading and unloading phases under different amplitude of pressure (maximum pressure, half of maximum pressure, and quarter of maximum pressure). At the velocity of 5 mm/s, the flat rod presses the samples at the velocity of 5 mm/s. (a-1) to (a-3) Results of EcoFlex samples with different thicknesses. (b-1) to (b-3) Results of VytaFlex samples with different thicknesses. (c-1) to (c-3) Results of MoldStar samples with different thicknesses. For all the samples, a bigger hysteresis ratio is shown with 1) larger pressure and 2) bigger shore hardness samples.

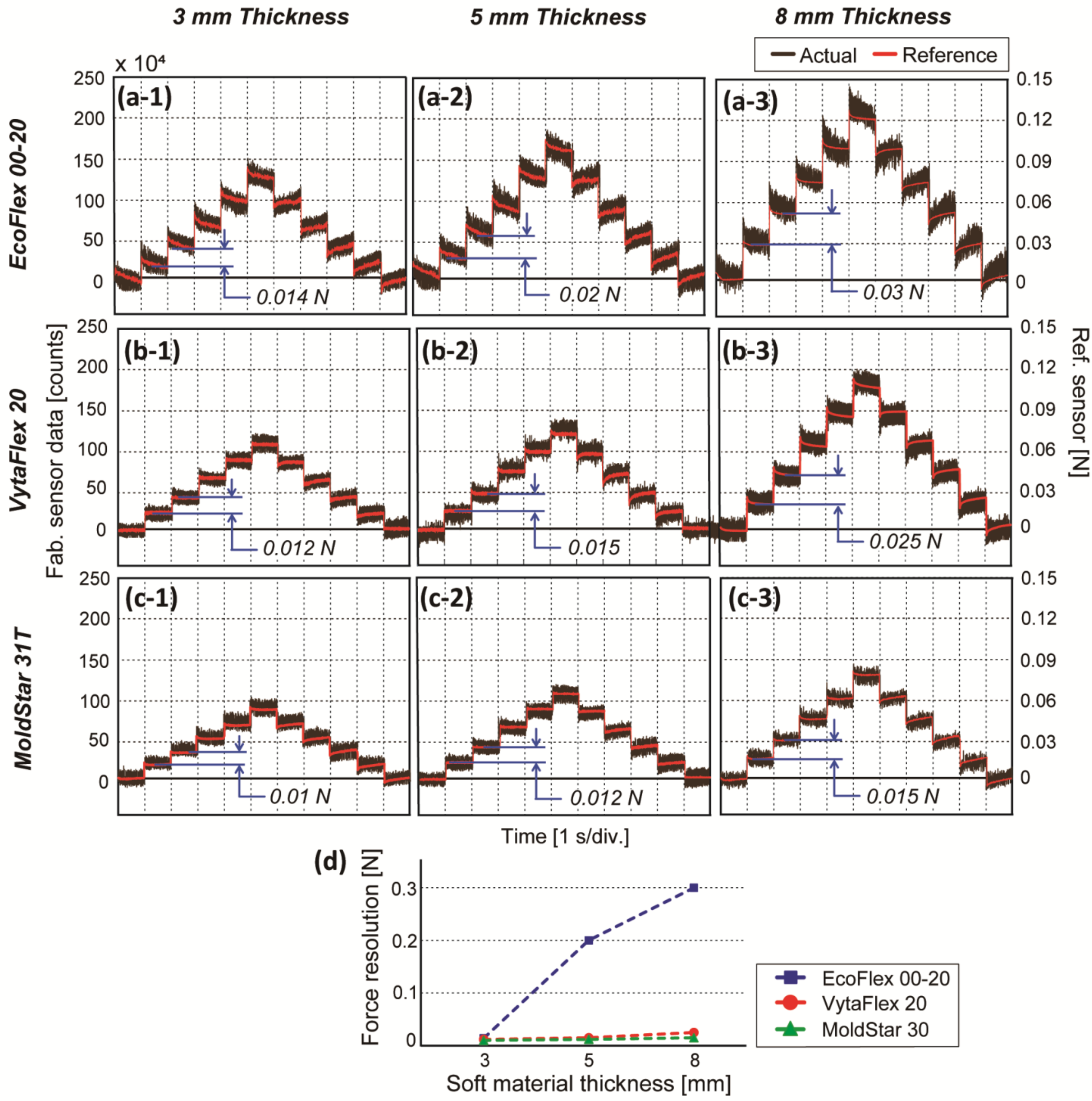

Supplementary Figure S4. Force resolution of nine samples. Flat rod presses the samples with the least detectable force in 1mm/s. (a-1) to (a-3) Results of EcoFlex samples with different thicknesses. (b-1) to (b-3) Results of VytaFlex samples with different thicknesses. (c-1) to (c-3) Results of MoldStar samples with different thicknesses. In contrast to the force measurement capacity, 1) bigger shore hardness samples and 2) thinner samples show bigger force resolution which can imply sensitiveness. (d) Organized force resolution graph with different material types and thicknesses.

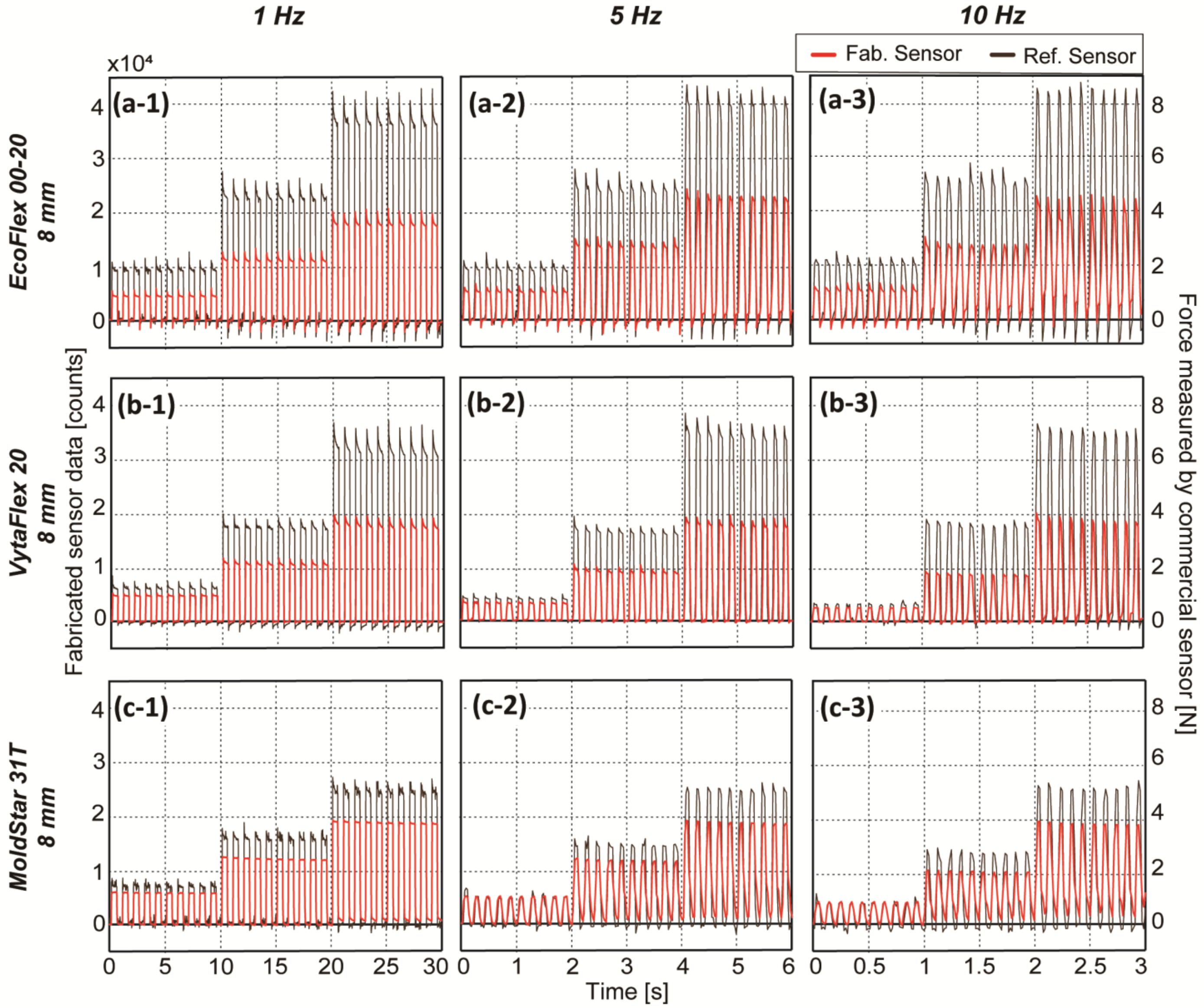

Supplementary Figure S5. Dynamic response under three different amplitudes of pressure with 8 mm thickness samples. Flat rod presses the samples in different frequencies (1, 5, and 10 Hz), and rod presses samples at three different distances 10 times in each experiment to make the different amplitude of pressure. (a-1) to (a-3) Results of EcoFlex samples with different thicknesses. (b-1) to (b-3) Results of VytaFlex samples with different thicknesses. (c-1) to (c-3) Results of MoldStar samples with different thicknesses. For the samples with smaller shore hardness (i.e., EcoFlex and VytaFlex), it tends to make stiff peaks when the loading phase initiates or finishes. This can imply these samples have a bigger restoring force. (a-3), (b-3) In higher frequencies with bigger pressure, they lack restoring power and require more recovery time than 0.2 s (i.e., 5 Hz). (c-1) The sample with bigger shore hardness hardly reaches zero even at the low frequency, which means it has less restoring power and requires more recovery time than 1 s (i.e., 1 Hz).
